# Dermoscopic Monitoring of Response to Intense Pulsed Light in Rosacea: A Case Report

**DOI:** 10.5826/dpc.1003a58

**Published:** 2020-06-29

**Authors:** Ajay Deshapande, Balachandra S. Ankad

**Affiliations:** 1 Dermatology, Pune, Maharashtra, India; 2 Department of Dermatology, S. Nijalingappa Medical College, Bagalkot, Karnataka, India

**Keywords:** rosacea, dermoscopy, intense pulsed light, monitoring

## Introduction

Rosacea is a chronic relapsing inflammatory skin disorder mainly affecting the central part of the face. Clinically, it is characterized by facial redness and erythematous papules and pustules. Due to overlapping features with other skin diseases, such as contact dermatitis, seborrheic dermatitis, acne vulgaris, cutaneous lupus, and carcinoid syndrome [1], the differential diagnosis is important. Therefore, dermoscopy may provide additional features to improve the recognition of rosacea, including polygonal vessels with superficial scales and follicular plugs [2]. We report herein the dermoscopic pattern of a patient with rosacea and variations after systemic and intense pulsed light (IPL) therapy.

## Case Presentation

**Figure 1:**
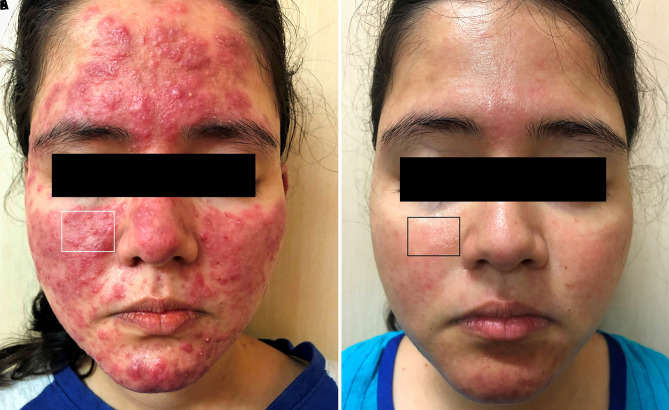
(A) Clinical image of a 22-year-old patient with a papulopustular rosacea before treatment. (B) Clinical image of the same patient showing the improvement of rosacea after systemic and intense pulsed light treatment. Boxes in both images represent the target areas where dermoscopy was performed.

**Figure 2:**
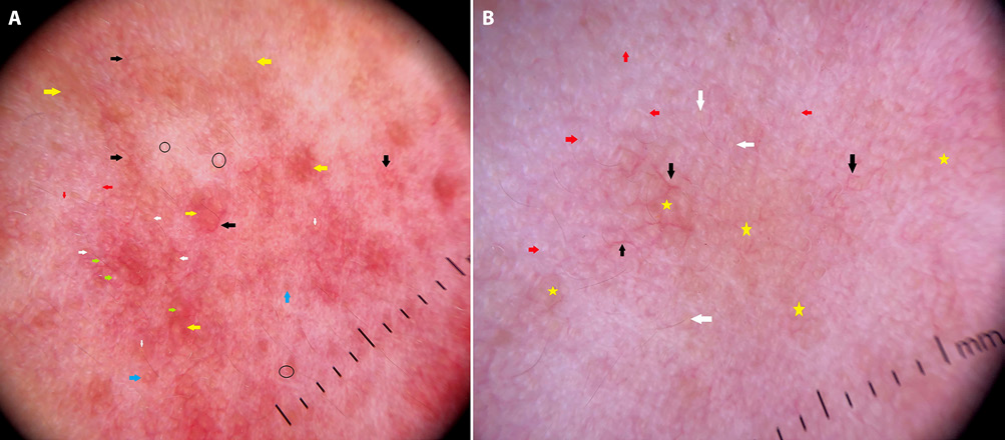
(A) Dermoscopy before treatment, showing brownish yellow areas (yellow arrows), linear vessels in polygonal pattern (black arrows), dilated follicles (blue arrows), follicular plugs (white arrows), ill-defined white rosettes (red arrows), and nonspecific scales (black circles). (B) Dermoscopy after treatment, showing a reduction in vascular (black arrows) and follicular structures (white arrows), compared with pretreatment picture. Brownish yellow areas (yellow star) and white streaks (red arrows) are also decreased. Polarized mode, magnification ×10.

A 22-year-old woman with Fitzpatrick skin type IV presented to our clinic with a 3-month history of intense red lesions of the face. Clinical examination revealed well-defined erythematous-edematous plaques covered with tiny papules, pustules, and a few nodules (Figure 1A). The corresponding dermoscopic aspect was characterized by polygonal linear vessels, follicular plugs, brownish yellow areas, scales, follicular pustules, dilated follicles, and irregular and ill-formed rosettes (Figure 2A). Based on these clinical and dermoscopic features, a diagnosis of papulopustular rosacea was made. Ivermectin 12 mg once a week and minocycline 100 mg once a day were given for 2 weeks. The patient was advised to apply broad-spectrum sunscreen. Immediately after, the first session of IPL treatment was performed. IPL was then repeated every week for 6 weeks. The vascular probe, with a wavelength spectrum of 550–1,100 nm, was used and the patient received 6 passes in single pulse mode at a fluence of 12 J/cm^2^ followed by 6 passes in continuous mode of 7.1 J/cm^2^.

Clinical and dermoscopic improvement after treatment is reported in Figures 1B and 2B. Dermoscopic patterns of rosacea have been described in the current literature [2]. These dermoscopic patterns were observed in the case described herein. However, we detected an additional brownish hue in the context of yellowish areas, probably due to the dark skin color of the patient. Furthermore, rosettes were ill-formed and ill-defined, resembling white shiny streaks in arcuate, short linear structures.

## Conclusions

Systemic treatment and IPL therapy are good options in rosacea [1]. IPL ablates dilated dermal vessels and helps in collagen remodeling and improving skin texture [1]. *Demodex*, which is commonly found in rosacea, is light- and heat-sensitive and known to aggravate rosacea during IPL treatment [1]. To prevent this reaction and to treat the papulopustular component, 2 doses of systemic ivermectin 12 mg weekly and minocycline 100 mg daily are advisable before initiating IPL therapy.

Monitoring the treatment response in rosacea is an important step in the management of the skin disease. In this case, the effectiveness of a combination of systemic and IPL treatments in rosacea was monitored by dermoscopy. We noticed significant improvement of vascular and follicular structures, scales, brownish yellow areas, and white streaks. Hence, dermoscopy is useful in the assessment of treatment in rosacea. Since dermoscopy is a rapid, easy, and noninvasive examination, it can be used in the clinical practice for the diagnosis of rosacea as well as for treatment monitoring.

## References

[1] Papageorgiou P, Clayton W, Norwood S, Chopra S, Rustin M (2008). Treatment of rosacea with intense pulsed light: significant improvement and long lasting results. Br J Dermatol.

[2] Errichetti E, Stinco G (2016). Dermoscopy in general dermatology: a practical overview. Dermatol Ther (Heidelb).

